# Orbital hopping maneuvers with two Astrobee free-flyers: Ground and flight experiments

**DOI:** 10.3389/frobt.2022.1004165

**Published:** 2022-11-25

**Authors:** Stephen Kwok-Choon, Jennifer Hudson, Marcello Romano

**Affiliations:** ^1^ Spacecraft Robotics Laboratory, Department of Mechanical and Aerospace Engineering, Naval Postgraduate School, Monterey, CA, United States; ^2^ Department of Mechanical Engineering, California Polytechnic State University, San Luis Obispo, CA, United States; ^3^ Department of Mechanical and Aerospace Engineering, Politecnico di Torino, Turin, Italy

**Keywords:** Astrobee, Astrobatics, space robotics, orbital hopping, ISS experiments

## Abstract

Dynamic hopping maneuvers using mechanical actuation are proposed as a method of locomotion for free-flyer vehicles near or on large space structures. Such maneuvers are of interest for applications related to proximity maneuvers, observation, cargo carrying, fabrication, and sensor data collection. This study describes a set of dynamic hopping maneuver experiments performed using two Astrobees. Both vehicles were made to initially grasp onto a common free-floating handrail. From this initial condition, the active Astrobee launched itself using mechanical actuation of its robotic arm manipulator. The results are presented from the ground and flight experimental sessions completed at the Spacecraft Robotics Laboratory of the Naval Postgraduate School, the Intelligent Robotics Group facility at NASA Ames Research Center, and hopping maneuvers aboard the International Space Station. Overall, this study demonstrates that locomotion through mechanical actuation could successfully launch a free-flyer vehicle in an initial desired trajectory from another object of similar size and mass.

## 1 Introduction: robotic hopping

Space-based activities are expected to increase with national and commercial entities, exploring how to access and utilize resources beyond Earth (Coto et al., 2021) Haws et al. (2019) (Edwards et al., 2021) (Swaminathan and Malhotra, 2021). Robotic vehicles can assist by performing tasks such as observation and measurement that would otherwise be time-intensive, laborious, and repetitive. Of particular interest is the use of self-toss hopping maneuvers that allow a vehicle through mechanical actuation to launch from a given object or surface. Robotic hopping with mechanical actuation has been explored for search and rescue, exploration, and more recently on-orbit exploration of asteroid Ryugu with MINERVA II (Fiorini and Burdick, 2003) (Dubowsky et al., 2008) (Yoshimitsu et al., 2012) (Bai et al., 2012).

Robotic hopping can be used in applications by orbital service vehicles performing tasks such as surveying, data collection, and object manipulation. Systems and platforms intended for refueling, servicing, and support of longer space missions are under development (Davis et al., 2019). Examples of on-orbit servicing missions that have occurred or are about to occur include the mission extension vehicles by Northrop Grumman that performed rendezvous and docking with the Intelsat IS-901 and IS-1002 satellites in a geosynchronous orbit (Pyrak and Anderson, 2022), the Robotic Servicing of Geosynchronous Satellites (RSGS) program for servicing interaction and manipulation of client satellites (Saplan, 2022), and the OSAM-1 (formerly Restore-L) servicing mission that aims to refuel and reposition Landsat 7 in order to extend its operational duration (Coll et al., 2020). As orbital robotic servicing capabilities improve, robotic manipulators can provide a new, efficient means of locomotion for servicing vehicles operating on the client spacecraft. Mechanical actuation of the manipulator can be used to launch a servicing vehicle off another structure, thus reducing the amount of the propellant needed by the servicing vehicle and increasing its potential operational time. Hardware-in-the-loop kinematic and dynamic testbeds are used to validate and prepare systems prior to flight (Wilde et al., 2019).

Astrobatics is a research collaboration between the Spacecraft Robotics Laboratory (SRL) of the Naval Postgraduate School (NPS) and the Intelligent Robotics Group (IRG) facility at NASA Ames Research Center. Astrobatics has performed research in self-toss hopping maneuvers presented in the study by Kwok Choon et al., (2019) Kwok-Choon et al., (2020) Kwok-Choon et al., (2021) Watanabe et al., (2022), where the results from proximal and distal hopping maneuvers of Astrobee from ground and onboard the International Space Station (ISS) from a fixed handrail were published. Previous Astrobatics experiments explored hopping maneuvers from a fixed handrail, which was mounted on the deck or interior wall of the ISS.

The dynamics of orbital robotic hopping are based on the principle of conservation of momentum. A spacecraft actuates one or more robotic joints, which imparts a velocity on the spacecraft body and allows it to launch itself off a base structure. Thus, it achieves motion through electrical power, which is renewable in an orbit, instead of a chemical propellant, which is limited. Mathematically, orbital hopping can be divided into two fundamental cases: hopping from a base structure with very large mass and hopping from a base structure with mass that is close in magnitude to the robotic spacecraft itself. In the first case, which was tested in previous Astrobatics experiments (Kwok-Choon et al., 2021), the structure can be treated as a fixed base, and reaction forces from the hopping maneuver can be neglected. In the second case, which is considered in this study, the hopping maneuver imparts a velocity on the base structure, based on the law of action and reaction. A theoretical model of the dynamic hopping maneuver is presented in the study by Watanabe et al., (2022). In that study, equations of motion were developed for a hopping maneuver with two Astrobee vehicles, with zero initial linear and angular momenta. In comparison, this study complements (Watanabe et al., 2022) by going into further detail on the hardware-in-the-loop ground and flight experiments at NPS SRL, Ames IRG, and aboard the ISS. The NPS SRL and Ames IRG ground experiments were performed from June 2021 to January 2022, with the ISS sessions completed in November 2021 and February 2022, respectively. The results of the dynamic hopping maneuvers are included here, showing the active and passive vehicle relative states, with a discussion outlining observations and comparisons.

The research presented in this article was motivated in exploring dynamic motion from hopping maneuvers between objects of similar size and inertia. Hopping maneuvers with two Astrobees were completed, where both Astrobees initially grasped onto a free-floating common handrail, and the designated active Astrobee launched itself from the passive Astrobee system using mechanical actuation of its robotic arm manipulator. A series of hardware-in-the-loop experiments were performed with two floating spacecraft simulators on the granite table at the Naval Postgraduate School, followed by testing with two Astrobees at NASA Ames Research Center, and finally followed by a set of flight experiments onboard the ISS.

This article is composed of five sections and delves further into the dynamic hopping maneuvers of one Astrobee free-flyer launching from another of similar size and mass. First, the Astrobee free-flyer and its three-degree-of-freedom robotic arm are described. Next, the dynamic hopping maneuver of the active Astrobee launching from the passive Astrobee system is presented. In the next section, the ground test facilities at SRL and IRG are outlined. Finally, the results of ground and flight experiments from SRL, IRG, and onboard the ISS are described, with discussion and conclusions drawn from the results obtained.

## 2 Astrobee free-flyer

**FIGURE 1:**
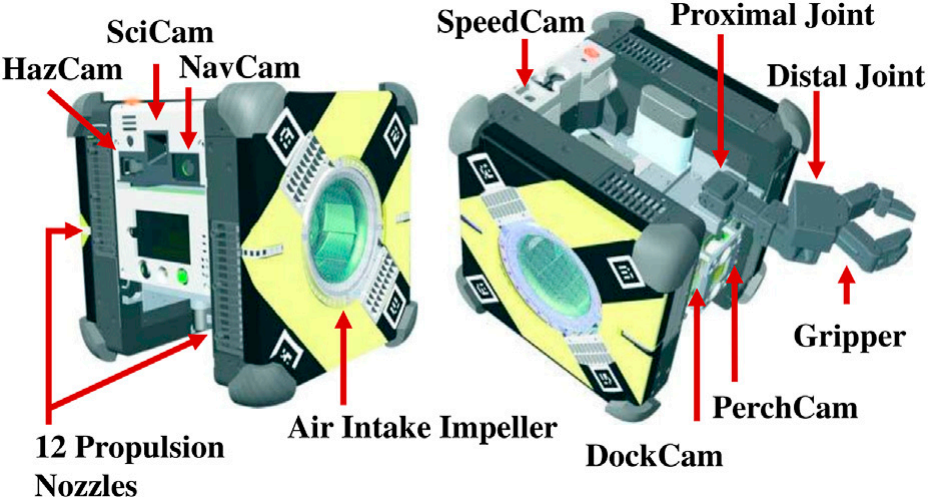
Illustration and annotation of the Astrobee free-flyer vehicle (Lee et al., 2018).

Astrobee is a free-flying robotic system designed to support crew activities and serve as a research platform onboard the ISS (see Figure 1). The Astrobee free-flyers are shaped like cubes 32 cm wide and equipped with two mounting bays for guest science hardware payloads; the examples include the Stanford gecko-adhesive gripper (Cauligi et al., 2020) and the SoundSee acoustic sensor (Bondi et al., 2022). The Astrobatics project has been of particular interest to the utilization of Astrobee’s three-degree-of-freedom robotic perching arm, composed of a proximal joint, distal joint, and gripper end-effector. The robotic arm has an actuation command range for the proximal joint [−30°, 90°] and distal joint [−90°, 90°] (Park et al., 2017).

Astrobee is equipped with lithium ion rechargeable batteries which are charged while the vehicle is berthed to a docking station, 12 nozzles for propulsion through the use of two air intake impellers, and an array of onboard sensors (Hazcam, SciCam, Navcam, Speedcam, PerchCam, and DockCam) that allow it to sense and interact with its environment. In addition, Astrobee has three main processors: the low-level processor (LLP) that controls the propulsion system and runs the control software, the mid-level processor (MLP) for computer vision algorithms, and the high-level processor (HLP) that is primarily used for guest science software applications. Further description of Astrobee’s capabilities can be found within Smith et al. (2016) and Park et al. (2017).

## 3 Hopping maneuver

An orbital hopping maneuver takes place when a spacecraft uses a robotic manipulator to push or toss itself off another space structure. In a previous work (Kwok Choon et al., 2019), the equations of motion for orbital hopping were defined using a Lagrangian approach. If a hopping maneuver is performed off a structure with very large mass, such as the ISS, the structure can be treated as a fixed base with infinite inertia, and the linear and angular momenta of the robotic spacecraft are conserved after the instant of separation (Kwok-Choon et al., 2021).

However, many potential orbital hopping applications involve a base structure with mass comparable to the robotic spacecraft itself. In these cases, the base must be treated as a free-floating object with non-infinite inertia, and the total linear momentum, *P*, and angular momentum, *L*, of the system (the robotic spacecraft and the base structure together) are conserved. For a system consisting of *n* links, the momenta are described by

$$P\left(t\right)=\overset{n}{\underset{i=1}{\sum }}m_{i}v_{i},$$ (1)

$$L\left(t\right)=\overset{n}{\underset{i=1}{\sum }}\left(I_{i}\omega_{i}+r_{i}\times m_{i}v_{i}\right),$$ (2)

where *n* is the number of rigid links of the complete system (including base structure, manipulator, and robotic spacecraft body), *m* is the mass of each link, ***v*** is the velocity of the center of mass of each link, *I* is the mass moment of inertia of each link about its center of mass, and *r* is the position of the center of mass of each link. The center of mass of the complete free-floating system can be treated as the origin of an inertial frame, in which the vector quantities are defined. If the system is initially at rest, *P*(*t*) = 0 and *L*(*t*) = 0 for all time *t*, the base and robotic spacecrafts will move with equal and opposite momenta after separation. A method to characterize the active and passive Astrobee dynamic models is utilization of equations of motion based on the generalized Jacobian matrix (Wilde et al., 2018) (Watanabe et al., 2022).

**FIGURE 2:**
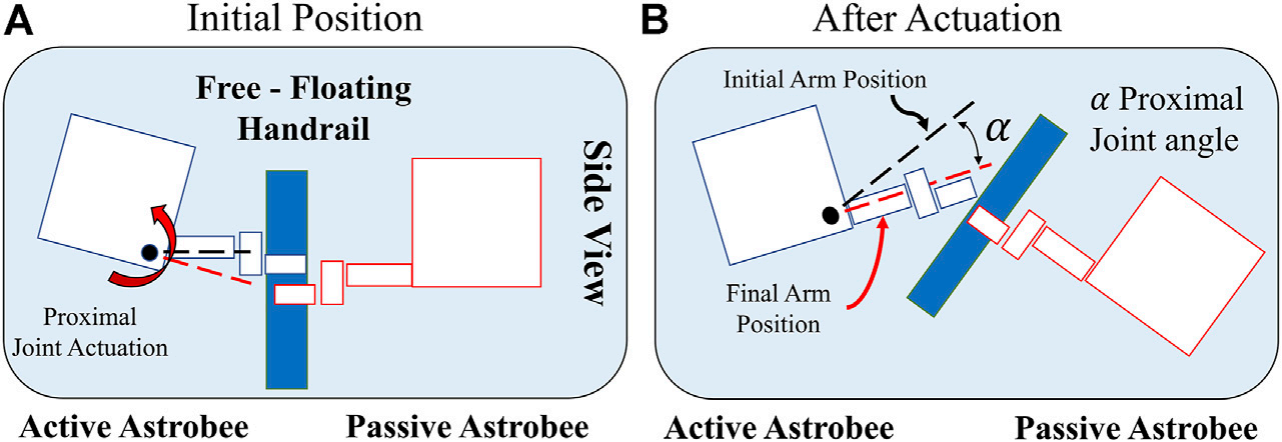
Astrobee hopping maneuver—side view: **(A)** Initial configuration with the proximal joint at initial arm position. **(B)** Illustration after actuation and gripper release.

In the orbital hopping experiments described here, the case of hopping off an object of comparable mass and inertia was investigated. The Astrobee hopping maneuver is illustrated (see Figures 2A,B), with both vehicles initially grasping onto a common free-floating handrail. For this series of tests, the free-floating handrail was secured to the passive Astrobee, where the grasp by the passive Astrobee on the handrail is equivalent to that of a rigid body composed of the Astrobee plus the handrail. From the start position (see Figure 2A), both the active and passive Astrobees were initially at rest. A self-toss hopping maneuver occurred with the actuation of the active Astrobee proximal joint, followed by gripper release. This series of actions imparts a change in angular and linear velocity of components on both sides of the release point, which upon gripper release caused both vehicles to travel on their respective free-flight paths (see Figure 2B).

Since the active and passive Astrobees are nearly identical in mass and inertia, it was expected that the two robots would separate with approximately the same speed in opposite directions. The main independent variable for these experiments was the joint angle at the instant of separation, which determines the direction of the velocity vector *v* in Eqs 1–2. Faster separation speeds were expected for cases in which *v* had a larger component, normal to the handrail. For each run, the ISS crew placed the system at a central start position, and the Astrobee operator then coordinated with the ISS crew for the release and start of the maneuver. During the hopping maneuver, each Astrobee collected its perceived trajectory and orientation state. The passive Astrobee was commanded to hold onto the free-floating handrail, with its robotic arm fully extended throughout the experiment, thereby providing a launch platform for the active Astrobee system. For each run, the active Astrobee robotic arm was commanded to actuate its proximal joint from an initial start angle, with gripper release set to occur upon reaching the desired final angle. A range of final angles were tested in order to evaluate self-toss maneuvers under different arm release conditions.

## 4 Ground test facilities

**FIGURE 3:**
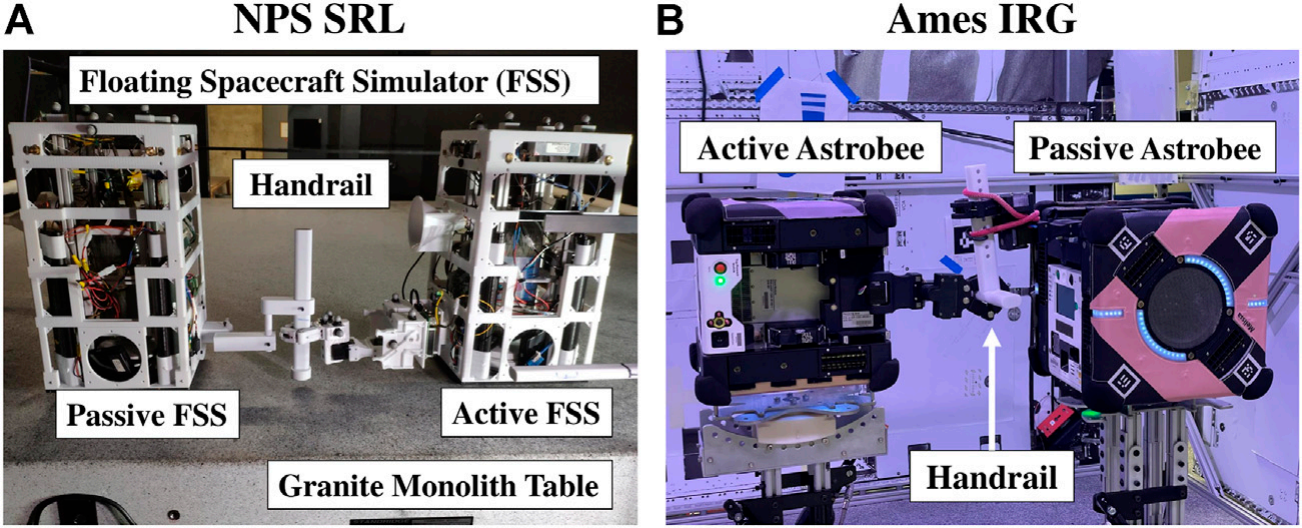
Experiment ground testing set up: **(A)** at NPS SRL and **(B)** at NASA Ames IRG (credit NASA).

In preparation for flight experiments aboard the ISS, a series of different runs were completed at the SRL and IRG test facilities (see Figure 3; Table 1). First, dynamic self-toss hopping maneuvers were developed and explored in the NPS SRL ground test facility. At SRL, two Floating Spacecraft Simulators (FSS) were used on a 4 m × 4 m granite monolith table (see Figure 3A). The active FSS was equipped with a replica Astrobee three-degree-of-freedom robotic arm (Komma, 2018), and the passive FSS had a 3D printed static representation of a mounted fixed arm. The FSS mounting base allowed for the Astrobee arm to be actuated in the plane of interest.

**TABLE 1:** Hopping maneuvers performed at SRL, IRG, and aboard the ISS.

	Case	Proximal joint (°)		Runs	${\dot{\bar{\alpha }}}_{\max}\left({}^{\circ}/s\right)$	Description
		Initial	Final			
SRL	A	−90	−30	3	6.9	Astrobee arm module attached and used on an active FSS
	B		−45	3	6.9	
	C		−60	3	6.9	
IRG	A	−20	0	3	5.4	Active Astrobee on its side with passive Astrobee upright
	B		15	3	5.4	
	C		45	3	5.4	
ISS	A	−20	0	7	5.4	R1,R2,R3,R4,R5,R6,R2,R3,R4 R1,R2,R3,R4,R2,R3 w/SAM
	B		15	4	3.6	
	C		45	3	5.4	
	D		15	3	5.4	

Each Astrobee vehicle is a cuboid of 32 cm × 32 cm and has a mass of approximately 10 kg (Bualat et al., 2018). This is comparable to the FSS vehicles, which have a footprint of 27 cm × 27 cm and a mass of 9.882 kg, with no payload attached (Zappulla et al., 2017). The similarity in size and mass of the different systems allowed for the dynamic comparison of expected motion during self-toss between the hopping maneuvers performed at SRL NPS and IRG NASA Ames.

Initial maneuver selection utilized full extension of the proximal joint on the active FSS. A series of experimental runs at NPS SRL were completed. However, it was determined that a large initial angle could potentially cause unintended contact of the active and passive Astrobee vehicles. Thus, to ensure no incidental collision between the active and passive Astrobee vehicles during ground and flight tests, the initial and final angles selected for the dynamic hopping maneuvers at NASA Ames IRG and aboard the ISS were set to a subset of the joint range (see Table 1).

After initial testing and demonstration of two-vehicle self-toss maneuvers at the SRL facility, a second round of ground testing was completed at the IRG granite laboratory using real Astrobee vehicles on floating platforms (see Figure 3B). To actuate the Astrobee proximal joint with motion parallel to the granite table, the active Astrobee had to be mounted on its side. Due to hardware mounting limitations, only the active Astrobee could be mounted on its side, and the passive Astrobee was mounted in the upright orientation. To enable the testing and necessary flight preparation to proceed, a T-bar handrail was used, which was securely mounted to the passive Astrobee arm through the use of a red bungee cord (see Figure 3B) to prevent slipping. In comparison, onboard the ISS, the two Astrobees were mounted to a free-floating, linear handrail, with both Astrobees in the upright orientation. The passive Astrobee gripper was secured to the handrail with a Kapton tape. The orange film tape is shown in Figure 10B, on the passive Astrobee’s gripper end-effector.

## 5 Results

In Table 1, the summary of the ground and flight test results is outlined. The ISS cases A, B, and C were similar to the runs performed at the NASA Ames IRG test facility with actuation from [−20° to 0°, 15°, and 45°], respectively. Case D involved actuation from [−20° to 15°], followed by a free-flight trajectory, and then with the command of Stop All Motion (SAM) to activate its impellers to bring Astrobee to rest. From the study by Coltin (2019), the maximum angular velocity of each joint of the Astrobee’s robotic arm was defined at 6.9 (°/*s*) [0.12 (rad/s)]. The recorded angular velocity of the proximal joint, ${\dot{\bar{\alpha }}}_{\max}$ , from experiments in SRL, IRG, and aboard the ISS is presented in Table 1. During each hopping maneuver, the joint velocity ramped up during actuation to reach a peak angular velocity; the values of ${\dot{\bar{\alpha }}}_{\max}$ reported in Table 1 are the average peak velocity across all runs of each case.

It is to be noted that the SRL Astrobee arm was commanded to actuate to the documented angular velocity (Coltin, 2019); however, during subsequent testing at NASA Ames IRG and aboard the ISS, the Astrobee arm was found to actuate at a slower angular velocity. From experimental observations, the maximum joint velocity during actuation was relatively constant within each respective set (SRL, IRG, and ISS) of experimental runs.

### 5.1 Ground experiments at NPS SRL and NASA Ames IRG

**FIGURE 4:**
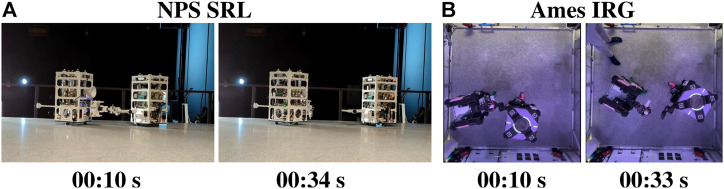
Experiment ground testing: **(A)** at NPS SRL and **(B)** at NASA Ames IRG (credit NASA).

The results from the NPS SRL and NASA Ames IRG ground experiments that were conducted for the experimental hopping maneuvers are outlined (see Figure 4). An example of the ground testing runs that were performed at NPS SRL is depicted (see Figure 4A), with an illustration of an experimental run that was performed at NASA Ames IRG (see Figure 4B).

#### 5.1.1 NPS SRL ground experiments

**FIGURE 5:**
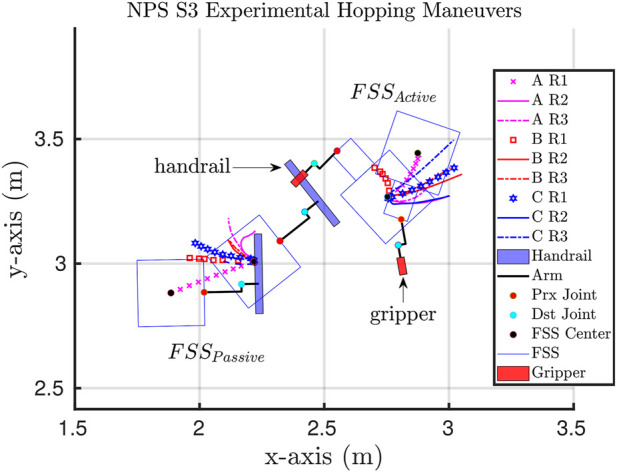
Illustration of ground experiments at NPS SRL, with the *FSS*_*Active*_ grasping onto the handrail held by the *FSS*_*Passive*_ as the initial start condition.

The NPS ground experiments were conducted with two floating spacecraft simulator (FSS) vehicles, where the active FSS was commanded to actuate its three-degree-of-freedom robotic arm and perform the hopping maneuvers from the handrail held by the passive FSS vehicle. An illustration of the FSS and the self-toss hopping maneuvers is shown in Figure 5, with the trajectories of cases A, B, and C tracked for each run.

**FIGURE 6:**
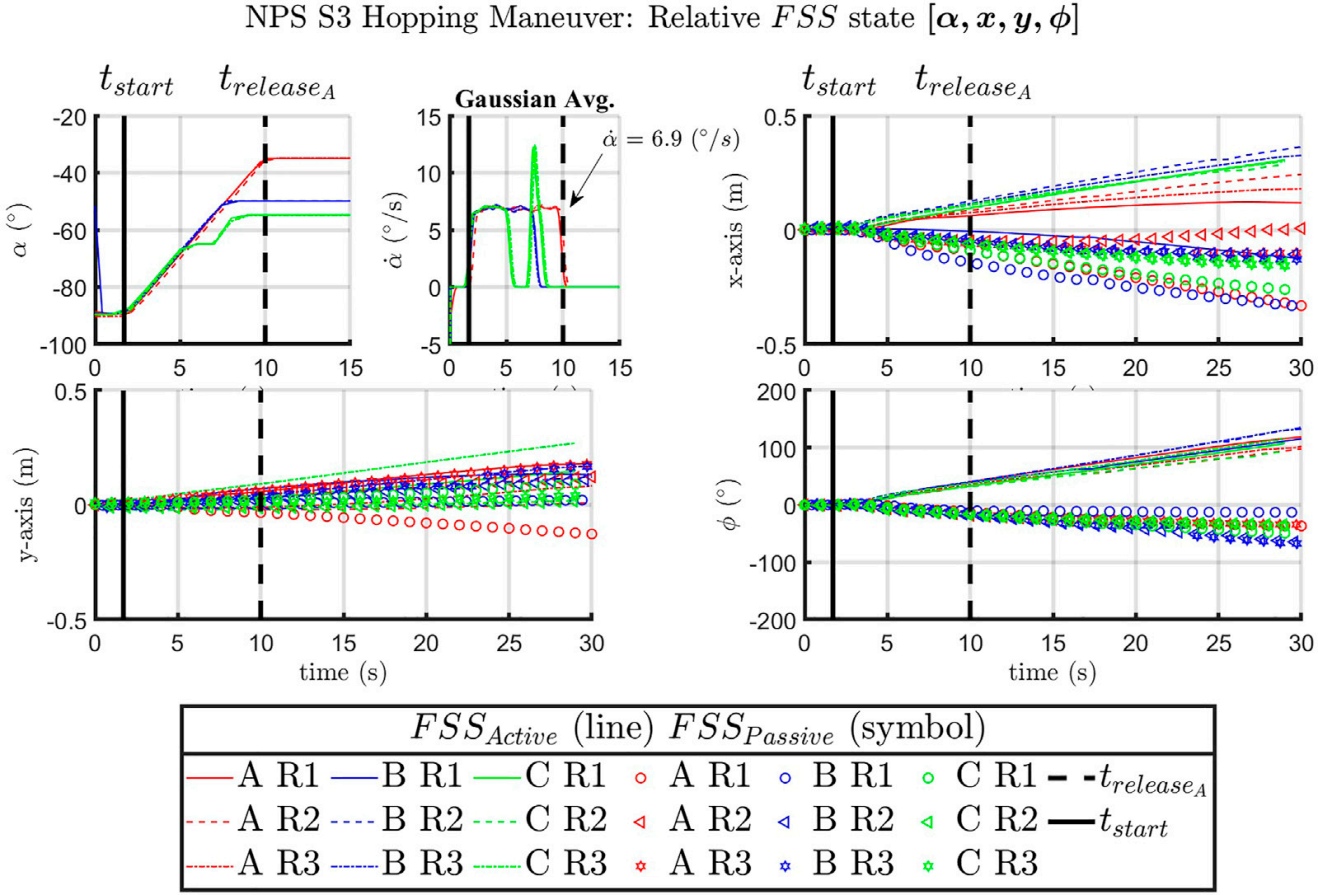
SRL [*α*, *x*, *y*, *ϕ*] for the *FSS*_*Active*_ vehicle over time. Time markers indicate *t*_*start*_ of proximal joint actuation and $t_{{release}_{A}}$ for case A.

Figure 5 was created by synchronizing the SRL runs to a common start position and orientation. Arbitrarily, run AR1 was chosen as the reference start position, thus allowing for the trajectories of all runs to be overlaid and compared. Each run had the same initial proximal angle for the active FSS, −90°, with varying final angles [−30°, −45°, −60°]. Figure 6 is an illustration of the three cases composed of nine respective runs, where the proximal joint was commanded to actuate from 20° to 0°, 15°, and 45°.

Figure 6 is a summary of the SRL hopping maneuvers from all case runs, where *α* represents the active Astrobee proximal joint angle, with $\dot{\alpha }$ the angular velocity of the active Astrobee proximal joint, and [*x*, *y*] displacement is the tracked position of the FSS, with the heading denoted by the angle, *ϕ*, in the z-axis. From Figure 6, the x- and y-displacement *vs.* time plots depict the trajectory of the *FSS*_*active*_ (line) compared to the *FSS*_*passive*_ (symbol) vehicle. After separation, the active and passive FSS vehicles are shown to move in approximately opposite directions. A similar effect is present in the orientation *ϕ* *vs.* time plot, which shows that the two vehicles have opposite rotations after separation.

It should be noted that the time at which the arm was commanded to move was synced in post-processing, *t*_*start*_ = 1.71 s, to allow for comparison and analysis of the respective datasets. This allowed for the time of release for case A runs to be synced at $t_{release_{A}}$ = 10.0 s. The angular velocity, $\dot{\alpha }$ , of the proximal joint was calculated from the derivative of the recorded joint position, *α*, and a smoothing Gaussian moving average was implemented to reduce the signal noise. The average maximum sustained angular velocity during joint actuation, $\dot{\bar{\alpha }}$ , was found to be 6.9°/*s* for the NPS hopping maneuvers.

#### 5.1.2 NASA Ames IRG ground experiments

In preparation for flight experiments, maneuvers of interest were verified on the NASA Ames IRG granite table with two Astrobees (see Figure 4B). While the orientation of the passive and active Astrobees is different than the expected configuration in the ISS session due to hardware limitations as described in Section 4, a full system test to verify code functionality was completed.

**FIGURE 7:**
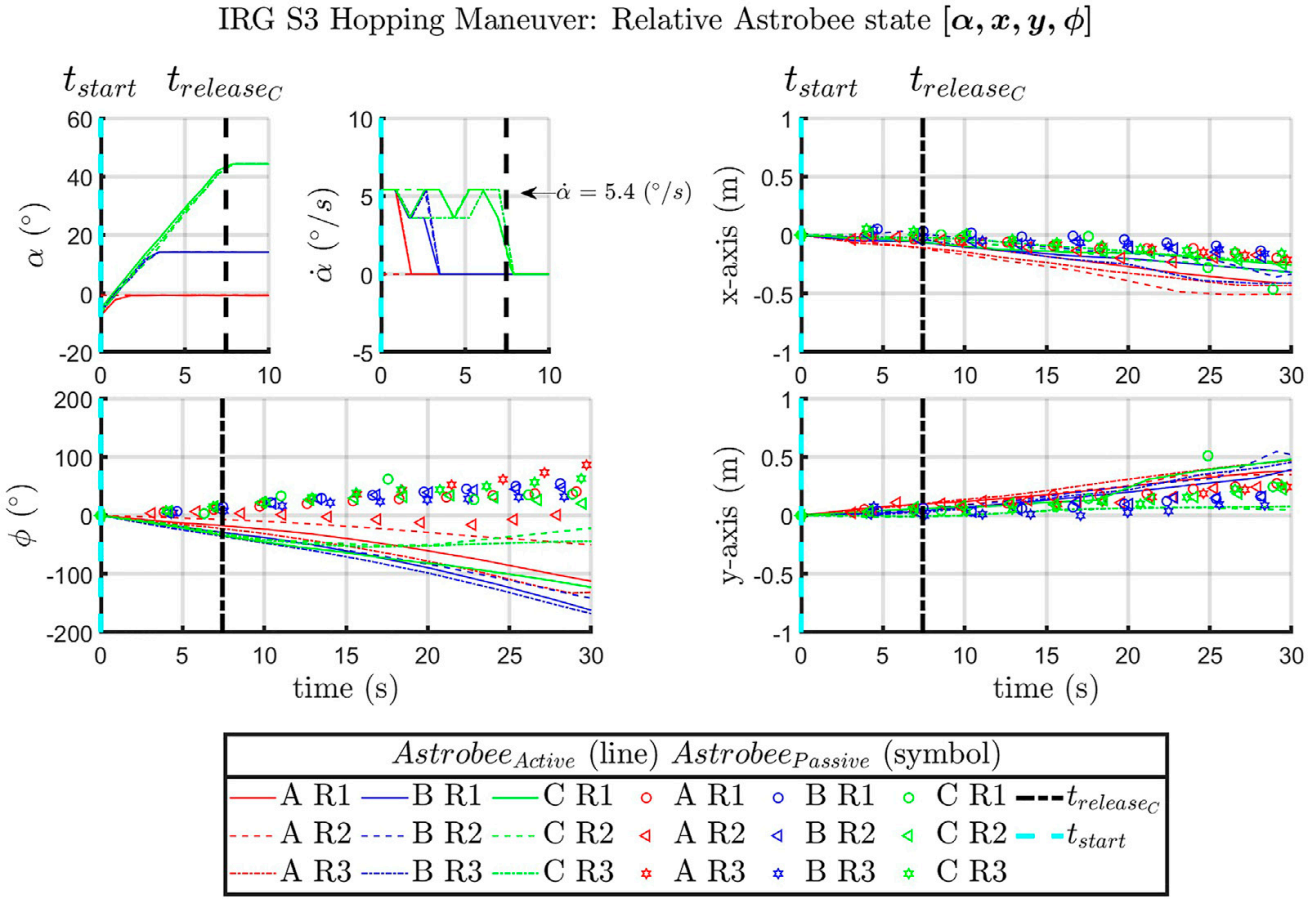
IRG [*α*, *x*, *y*, *ϕ*] for the *Astrobee*_*Active*_ vehicle over time, with the time markers for *t*_*start*_ of the proximal joint actuation.

The hopping maneuvers performed at NASA Ames IRG were carried out to validate and ensure that the commands and maneuvers of interest were possible with Astrobee. A summary of the recorded state, [*α*, *x*, *y*, *ϕ*], is presented with the actuation of the robotic arm manipulator proximal joint, *α*, xy-displacement, and planar orientation of *Astrobee*_*Active*_ (see Figure 7). The magnitude of planar xy-displacement in the Ames IRG datasets appeared to compare well with the NPS SRL experiments.

**FIGURE A1:**
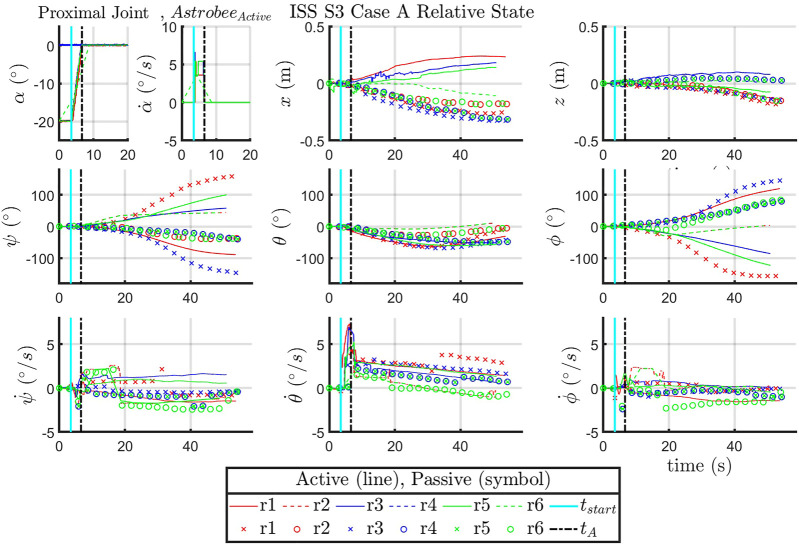
ISS: State data collected case A.

**FIGURE A2:**
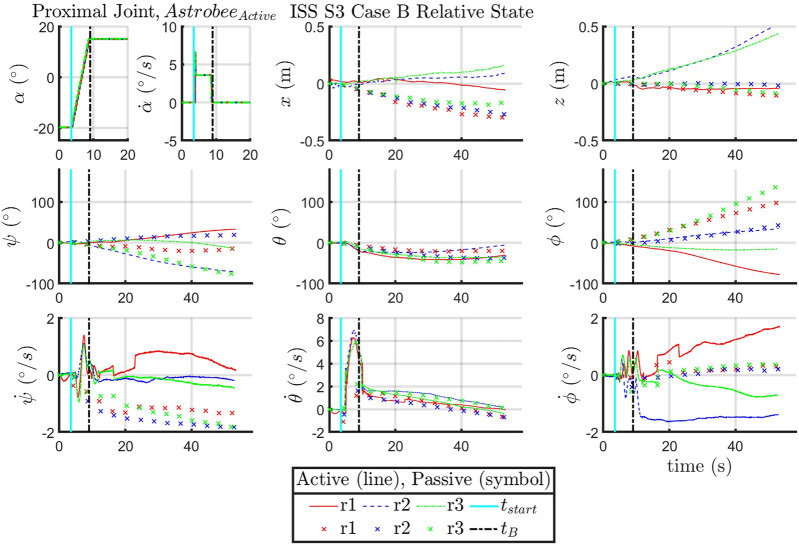
ISS: State data collected case B.

**FIGURE A3:**
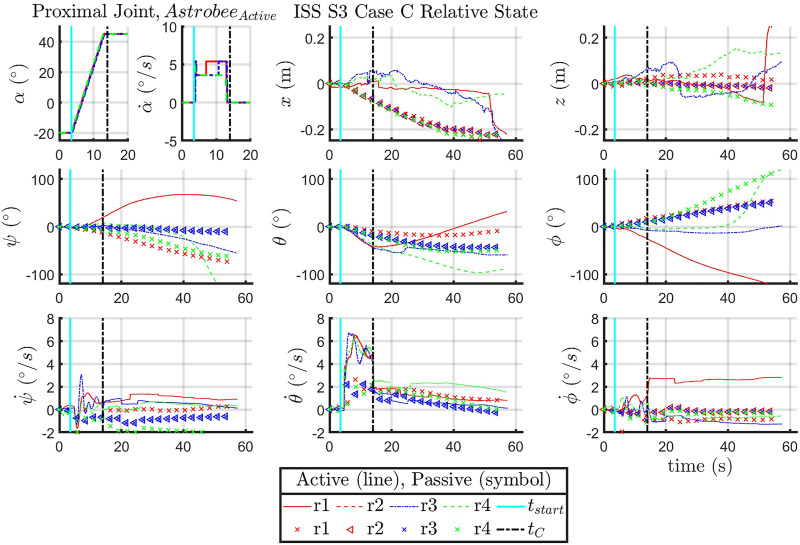
ISS: State data collected case C.

**FIGURE A4:**
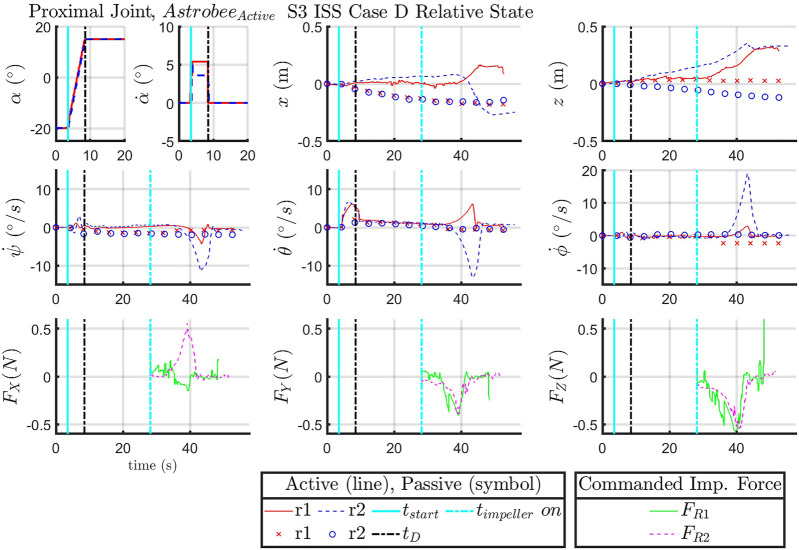
ISS: State data collected case D.

In post-processing, the command developed to start recording was found to overlap with joint actuation. This led to an unintended truncation in the start of data collection, where *t*_*start*_ was supposed to be at −20° (see Figure 7). This was resolved prior to the ISS flight experiments with the inclusion of a delay prior to the joint actuation command. (see Figures A1–A4).

### 5.2 International Space Station flight experiments

**FIGURE 8:**
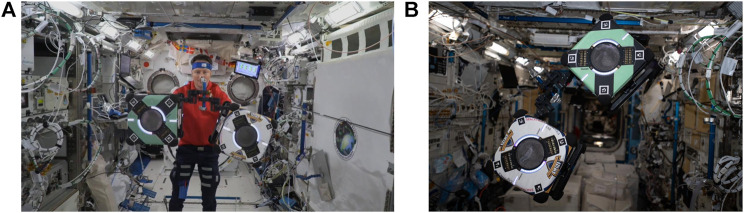
Hopping experiments onboard the ISS; **(A)** image taken from the observation camera during the experiment (credit NASA). **(B)** Hopping maneuver configuration (credit NASA) (Garcia, 2021).

**FIGURE 9:**
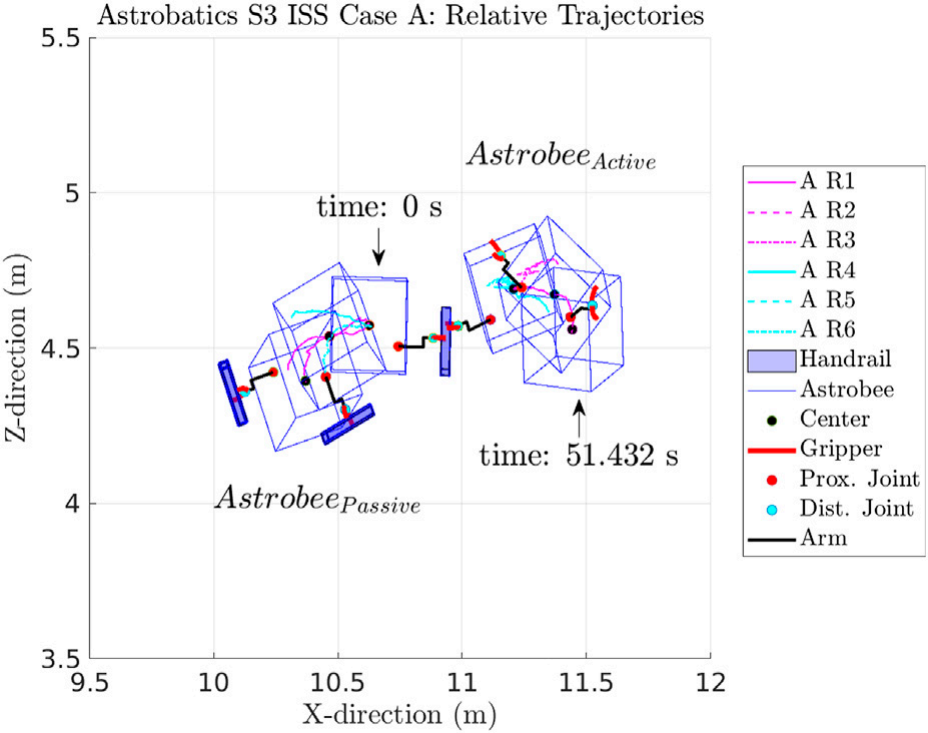
Illustration of the six case A runs that were completed with the relative trajectories of each run aboard the ISS.

The hopping maneuvers aboard the ISS, similar to the ground experiments performed at SRL and IRG (see Figures 8A,B), had the active Astrobee launch from the passive Astrobee system, where both initially grasped onto a common free-floating handrail. Figure 8A shows how the ISS crew placed the overall system prior to each experimental run. An illustration of all six successfully recorded ISS case A hopping maneuvers is shown in Figure 9, where the active Astrobee performed the hopping maneuver from an initial angle of −20° to a final angle of 0°.

A summary of the collected dynamic hopping maneuvers is shown in Figures A1–A4, with plots from each of the respective cases performed (see Figures A1–A4). In each figure, the initial position and orientation of the handrail was adjusted to a common baseline, in order to compare the resulting trajectories. The ISS hopping maneuvers of the active from the passive Astrobee in cases A, B, C, and D were completed in two sessions aboard the ISS. The first session was in November 2021, with the majority of the runs, and successful tests were completed in February 2022.

From Figure A1, a summary of the data collected during case A is depicted. In particular, the proximal joint $\left[\alpha ,\dot{\alpha }\right]$ position and angular velocity is displayed in conjunction with the displacement [*x*, *z*], angular position [*ψ*, *θ*, *ϕ*], and angular speed $\left[\dot{\psi },\dot{\theta },\dot{\phi }\right]$ . The time, *t*_*start*_, outlines when the proximal joint starts actuation for the maneuver, and the time, *t*_*A*_, is when the joint reached the desired angle followed momentarily by the commanded actuation release of the handrail. During actuation of the proximal joint, there appears to be an increase in the angular speed of the active Astrobee, $\left[\dot{\psi },\dot{\theta },\dot{\phi }\right]$ . The observed momentary increase in angular speed of the active Astrobee prior to release could be attributed to a change in the center of rotation of the active Astrobee, as it rotates about its proximal joint prior to release and rotates about its center of mass after release. The drop in the rotation rate after *t*_*A*_ could be due to energy loss during gripper release and an inbuilt safety function which reduces the joint velocity prior to gripper actuation. At gripper release, the active and passive systems separate as shown within the cases A, B, C, and D datasets found in Figures A1–A4.

Plots of the Astrobatics ISS experiments from cases B and C are provided (see Figure A2 and Figure A3). Similar to Figure A1, the passive and active Astrobee states during the hopping maneuvers are depicted. The time at which the Astrobee arm was commanded to actuate is shown as *t*_*start*_, with the moment that the desired proximal angle, *α*, was reached by time [*t*_*B*_, *t*_*C*_]. The release of the active from the passive system correlates to when the gripper release occurred as shown by the momentary spike in the angular rate, $\left[\dot{\psi },\dot{\theta },\dot{\phi }\right]$ , during the release of the active from the passive Astrobee.

Case D (see Figure A4) involved hopping maneuvers followed by the activation of the impellers to bring the active Astrobee to rest through Stop All Motion (SAM). The active Astrobee arm proximal joint started at an initial angle, −20°, and was then commanded to the desired angle of 15° at release. At time, *t*_*impeller*_, the impellers of the active Astrobee were turned on with the commanded impeller force [*F*_*R*1_, *F*_*R*2_] opposite the measured velocity. As shown, the activation of the impellers caused a momentary increase followed by stabilization in the angular rate and linear position of the active Astrobee free-flyer.

### 5.3 Observations

Overall, dynamic hopping maneuvers were successfully completed in ground and flight tests at NPS SRL, NASA Ames IRG, and aboard the ISS. Dynamic hopping maneuvers through mechanical actuation can be used as a method of initial locomotion for free-flyer vehicles near or on large space structures. Such maneuvers allow a free-flyer such as Astrobee to launch itself from a given object in an initial trajectory. In each case, as expected, the free-floating passive Astrobee was launched in the direction opposite to the active Astrobee. For cases A, B, and C as the final release angle increased, the overall x-displacement between the active and passive Astrobees decreased (see Figures A1–A3).

It is to be noted that sensor noise and the ability of each Astrobee to reliably determine its respective localization state (position and orientation) were important factors in capturing the recorded state of each vehicle. To plot and compare the trajectory datasets for each case, it was necessary to set a reference run within each case and then compare all other runs of that case to that set. In a similar vein, to compare the magnitude of the displacement of the passive and active free-flyer vehicles from dynamic hopping maneuvers, each set of trajectories were “zeroed” to the reference run in each case (see Figures A1–A4).

**FIGURE 10:**
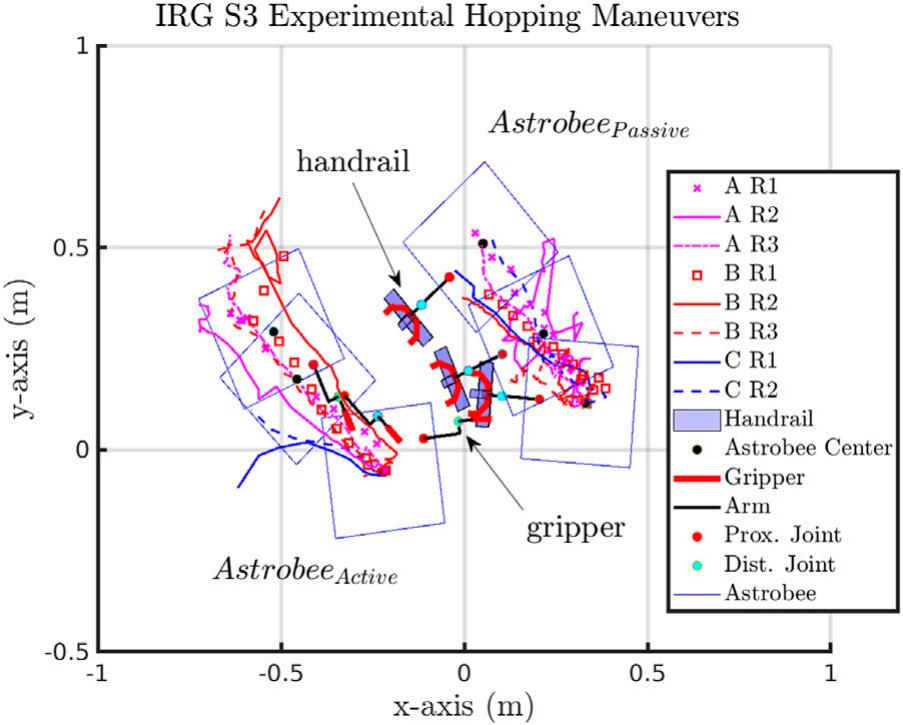
Illustration of the ground experiments at NASA Ames IRG, with the active Astrobee actuating its robotic arm in the plane and the passive Astrobee grasping the T-handrail in the upright orientation.

In comparison to the NPS experimental hopping maneuvers, the IRG and ISS experimental runs had irregular tracked trajectories. This could be due to the method of localization and determination of each vehicle’s position and orientation state [*x*, *y*, *z*, *ψ*, *θ*, *ϕ*]. During the NPS experiments, the FSS vehicles were tracked with a 10-camera Vicon tracking array mounted to the walls of the facility (vicon, 2022). During the IRG and ISS hopping maneuvers, Astrobee used its onboard sensors and cameras to determine its localization state (see Figure 10). The IRG granite table simulated the ISS environment (see Figure 4B) with reliance on tracking known features to allow Astrobee to determine its state. At the IRG, good feature tracking was possible when Astrobee observed features on the left, right, and rear walls. However, when Astrobee faced the open side of the table (near wall), there was poor tracking because of sparse features in the proximity of Astrobee. Hence, this would, in part, explain the irregular tracked trajectories observed in Figure 10, where the near wall was located at the top of the figure. To a similar extent, irregular jumps in the recorded trajectories from experiments performed aboard the ISS could also have been caused due to temporary loss of tracking features during each hopping maneuver (see Figures A1–A4).

The IRG granite table experiments provided validation and verification that the algorithms and hopping maneuvers developed would allow for Astrobee command aboard the ISS. Similar to the NPS experiments, the IRG experiments were constrained within a planar representation of the actuation and dynamic motion. The IRG experiments were allowed for the system and code testing of functionality, which was not previously present within previous experiments (Kwok Choon et al., 2019; Kwok-Choon et al., 2020; Kwok-Choon et al., 2021), such as the autonomous start and stop of data collection synced with actuation command and completion of the hopping maneuver, synchronous command and control of both the active and passive Astrobees at the same time, and sending command to one selected Astrobee at a time. The NPS and IRG test cases highlighted that hopping maneuvers of an active from a passive system of similar size and mass is a viable method to initiate locomotion and maneuvering.

In comparison, the IRG datasets appear to show a bias of drift for both the active and passive Astrobees in the -x-axis and +*y*-axis direction that does not seem to be as prevalent or consistent in the ISS cases A, B, C, and D datasets. The possible reason for the consistent drift in the IRG dataset could be due to possible preferential gradient on the granite table and related to test conditions. This is in direct comparison to the NPS experimental dataset trajectories that appear to be similar in separation post-gripper release of the active from the passive Astrobee vehicle, as shown in Figure 6.

## 6 Conclusion

Experimental hardware-in-the-loop results from ground and flight tests of orbital hopping maneuvers are presented. The active Astrobee free-flyer could successfully perform hopping maneuvers from a free-floating handrail held by a passive Astrobee. The experimental results agree with a theoretical model of orbital hopping, for the case of hopping from a free-floating base object with mass and inertia that are close in magnitude to the robotic spacecraft itself. The experiments demonstrated that this type of locomotion can be described by conservation of linear and angular momentum about the initial system center of mass; the active robot and the passive base are launched in opposite directions with opposite rotations.

The experimental results indicate that mechanical actuation can provide initial locomotion and propulsion of an orbital free-flyer from an object of similar size and mass. Applications of such hopping maneuvers could be used for on-orbit assembly, servicing, ferrying of construction materials, or transportation of sensors over larger space structures.

## Data Availability

The raw data supporting the conclusion of this article will be made available by the authors, without undue reservation.

## Nomenclature

***α***: Proximal joint angle
***ψ*, *θ*, *ϕ***: Euler angle orientation of the FSS and Astrobees.
$\overset{˙}{\psi },\overset{˙}{\theta },\dot{\phi }$: Euler angle rate of the FSS and Astrobees.
***FSS***: Floating Spacecraft Simulator
***IRG***: Intelligent Robotics Group
***ISS***: International Space Station
***NPS***: Naval Postgraduate School
**[*r*1, *r*2, *r*4, …*rn*]**: Recorded run of a particular case
***SAM***: Stop All Motion
***SRL***: Spacecraft Robotics Laboratory
***t*_*start*_**: Time at start of joint actuation
***t*_*A*_, *t*_*B*_, *t*_*C*_, *t*_*D*_**: Time at release of the gripper for each respective case
$t_{release_{\left(\;\right.}A,\left.C\right)}$: Time at release for cases A and C of NPS and IRG experiments, respectively
***x*, *y*, *z***: Cartesian position of FSS and Astrobees
